# Preventing Diseases by Inoculation

**Published:** 1881-07

**Authors:** Wesley Miller

**Affiliations:** New York


EDITOR OF JOURNAL OF COMPARATIVE MEDICINE:

### PREVENTING DISEASES BY INOCULATION.

EDITOR OF JOURNAL OF COMPARATIVE MEDICINE:

The recent report of the Department of Agriculture for 1880 on Contagious Diseases of Domestic Animals, especially with reference to pleuro pneumonia, the foot-and-mouth disease, and other kindred diseases, brings up the propriety of including in the researches concerning these contagious diseases the connection they bear to diseases in man. It would be well to investigate, for instance, if a preventive of scarlet fever is not to be found in the foot-and-mouth disease, and whether the purified virus of bovine tuberculosis cannot be applied, under certain conditions, to the human family, as in vaccination, as a preventive for phthisis pulmonalis. It is proven that diseases of domestic animals can be communicated to human beings, and, by vaccination of a purified virus, give protection for a specific disease.

If a preventive is discovered, that contains within itself a mild and effective means, that will give immunity, or lessen the severity of a disease that is contagious or not contagious, and applied as in vaccination, it is of immense importance to the human family. From practical experience in collecting and testing the animal or bovine virus from the calf for vaccination, as a preventive in small-pox, I find neither heat, cold, nor filtration changes the character of a virulent poison. But when cultivated in the systems of different animals, the virulence of the poison is taken away. It can then be used without any danger on healthy organisms, and will, in some diseases, certainly act as a preventive.

It is now well understood that the influence of one poison has the power of neutralizing or destroying the action of another. This is proven in vaccination for small-pox; and by the Negro, or Cholo, in Mexico, who inoculates himself with the poison of the rattlesnake, which renders him safe from the bite of all venomous animals. Trousseau perpetuated the records of the brave old pioneer of vaccination, Benjamin Jerty, who, long before Jenner, discovered the power of the cow-pox to produce immunity from small-pox, and applied the discovery to his own family.

The most difficult question that now confronts the medical and veterinary profession is that which relates to the prevention of disease. It is well said there is more of skill in preventing, or lessening, the severity of a disease, than in trying to cure it.

WESLEY MILLER, M.D.

NEW YORK, May 28th.

